# Total arterial revascularization in patients with acute myocardial infarction – feasibility and outcomes

**DOI:** 10.1186/s13019-017-0691-4

**Published:** 2018-01-05

**Authors:** Philippe Grieshaber, Lukas Oster, Tobias Schneider, Victoria Johnson, Coskun Orhan, Peter Roth, Bernd Niemann, Andreas Böning

**Affiliations:** 1 Department of Adult and Pediatric Cardiovascular Surgery, University Hospital Giessen, Rudolf-Buchheim-Str. 7, DE-35392 Giessen, Germany; 2 Department of Anaesthesiology, Sana Hospital Berlin-Lichtenberg, Berlin, Germany; 3 Department of Cardiology and Angiology, University Hospital Giessen, Giessen, Germany

**Keywords:** Acute myocardial infarction, Coronary artery disease, Coronary artery bypass grafting surgery, Revascularization, Total arterial revascularization

## Abstract

**Background:**

In acute situations such as acute myocardial infarction (AMI) with indication for coronary artery bypass grafting (CABG), total arterial revascularization (TAR) is often rejected in favour of saphenous vein (SV) grafting, which is assumed to allow for quicker vessel harvesting, a simpler anastomosis technique, and thus quicker revascularization and fewer bleeding complications. The aim of this study was to evaluate whether reluctance to apply TAR in AMI is still justified from a technical point of view in the current era and whether superiority of TAR results is also evident in emergency patients with AMI undergoing CABG.

**Methods:**

In this retrospective analysis of 434 consecutive patients undergoing CABG for AMI with either TAR or with a combination of one internal mammary artery and SV grafts between 2008 and 2014, procedural data, short-term and mid-term outcome were compared. Propensity score matching of the groups was performed.

**Results:**

After propensity score matching, 250 patients were included in the analysis (TAR group: *n* = 98; SV group *n* = 152). The procedural time (TAR group: 211 min vs. SV group: 200 min, *p* = 0.46) did not differ between the groups. Erythrocyte transfusion rates were higher in the SV group (76% vs. 57%; *p* < 0.001). Rates of re-exploration for bleeding did not differ. Thirty-day mortality rates were comparable (TAR group: 3.4% vs. SV group: 4.5%, *p* = 0.68). Kaplan-Meier analysis until 7 years postoperatively revealed a tendency for improved survival after TAR (75% vs. 62%; log-rank *p* = 0.12).

**Conclusion:**

TAR neither impairs rapid revascularization nor reduces its safety in patients with AMI. It may result in improved long-term outcome and should be preferred in the clinical setting of AMI.

## Background

The use of arterial grafts for coronary artery bypass grafting surgery (CABG), particularly bilateral internal mammary arteries (BIMA), is recommended due to the superior patency of these grafts compared with saphenous vein grafts (SV grafts) [1]. In real-world practice, however, the utilization of total arterial revascularization (TAR) lags behind these recommendations [2–5]. Reasons for reluctance to conduct total arterial CABG even in stable patients include the increased technical demand, the increased operation time, and fear of bleeding complications and impaired wound healing [6–8]. In patients undergoing CABG for acute myocardial infarction (AMI), large-scale data on TAR rates is limited, and rates ranging from 2 to 58% have been described [9, 10]. In the unstable situation of AMI, the above-mentioned arguments against total arterial CABG might play an even more important role for decision-making, as patients with AMI undergoing urgent or emergent surgery would be expected to benefit from short operation times and rapid revascularization afforded by use of venous grafting. Furthermore, AMI patients are frequently administered dual antiplatelet therapy (DAPT) preoperatively, resulting in increased risk of bleeding complications [11–13].

It is currently unclear whether these concerns about the use of TAR in patients with AMI are valid in the current era of surgical myocardial revascularization. Furthermore, the possible effect of total arterial CABG on long-term outcome in AMI patients has never been explicitly investigated.

## Methods

### Study population

We conducted a retrospective, single-centre study comparing patients undergoing total arterial CABG (total arterial revascularization group [TAR group]) or CABG with a combination of one internal mammary artery (IMA) and saphenous vein grafts (saphenous vein graft group [SV group]). Adult patients with a diagnosis of AMI (non-ST-segment elevation myocardial infarction [NSTEMI] or ST-segment-elevation myocardial infarction [STEMI]) within a period of 5 days or less before CABG without concomitant procedures (e.g. valve surgery) between 01/2008 and 12/2014 were included in the analysis. Patients with low cardiac output syndrome (LCOS) or cardiogenic shock at the time of surgery were excluded. The local ethics committee approved the study.

### Data collection, follow-up, definitions

Patients were identified according to the inclusion criteria from institutional patient records, and their baseline characteristics and perioperative data from the patient records and from data transferred to the nationwide quality assurance system (BQS Institute for Quality and Patient Safety, Hamburg, Germany) were analysed. Long-term follow-up was conducted via telephone interviews with the patients or their family physicians.

AMI was defined according to the Third Universal Definition of AMI [14]. The time of AMI was defined as the time of symptom onset. ‘Complete revascularization’ was defined using the concept of anatomical complete numeric revascularization’ (bypassing of all vessels ≥1 mm with hemodynamically relevant stenosis, as assessed by coronary angiography) [15]. We quantified the surgeon’s experience according to the years in practice since board certification as cardiac surgeon.

### Endpoints

We compared intraoperative parameters (duration of surgery, completeness of revascularization), perioperative need for invasive ventilation, perioperative transfusion requirements and bleeding complications, acute kidney injury as defined by KDIGO (Kidney disease: improving global outcomes) [16], sternal wound impairment requiring surgical therapy, postoperative duration of intensive care unit stay and hospitalization, as well as short- and mid-term survival between the groups.

### Management strategy

Patients who underwent cardiac catheterization for AMI are referred to our unit immediately after completion of the angiographic diagnosis and the heart team-based decision for CABG. The timing of surgery is determined by the surgeon on duty. CABG with the goal of complete revascularization is routinely performed on-pump with cardioplegic arrest using cold-blood cardioplegia (Buckberg) [17]. Acetylsalicylic acid is started 6 h postoperatively and continued lifelong at 100 mg/day. P2Y_12_ inhibitors are started on the first postoperative day and are continued for 12 months.

### Statistics

An inferential statistical analysis was performed using SPSS Version 24 (IBM, Armonk, NY, USA), GraphPad Prism version 6 software (GraphPad Software, Inc., La Jolla, CA, USA), and R version 3.1.2. Patient characteristics and outcomes were compared using Fisher’s exact test, Student’s t-test, or Wilcoxon-Mann-Whitney test, as appropriate. Continuous variables are presented as mean ± standard deviation (SD) unless stated otherwise.

In order to correct for potential confounding baseline parameters between the TAR group and the SV group, we carried out propensity score matching of the groups**.** Covariates included in the matching were age, gender, body-mass index, extent of coronary artery disease, preoperative left ventricular ejection fraction, diabetes mellitus (absence thereof, presence without insulin treatment, presence with insulin treatment), and EuroSCORE II. Nearest-neighbour matching in a 1:2 (TAR group vs. SV group) fashion was then performed. The maximum caliper between matched participants was set at 0.2. Long-term survival functions were determined using Kaplan-Meier estimation and compared using the log-rank test.

## Results

### Baseline data

A total of 434 patients were identified according to the inclusion criteria. Of these, 293 underwent CABG using a combination of one internal mammary artery and saphenous vein grafts, 3 underwent CABG with only vein grafts, and 138 underwent CABG using TAR. Baseline characteristics between the TAR group and the SV group differed significantly, with the TAR group having a lower proportion of female patients (17% vs. 29%; *p* = 0.011), a lower mean age (59 years vs. 71 years; *p* < 0.01), a lower rate of chronic kidney disease and a lower rate of patients with severely reduced left-ventricular ejection fraction (Table 1). Consecutively, the operative risk estimation using EuroSCORE II was lower in the TAR group than in the SV group (3.4% vs. 7.2%; *p* < 0.01) (Table 1). After propensity score matching, 250 patients (TAR group: *n* = 98, SV group: *n* = 152) remained in the analysis. The differences in baseline characteristics were eliminated (Table 1). All results described in the following refer to the matched groups. All patients received antiplatelet therapy before surgery, and 33% in the TAR group and 34% in the SV group (*p* = 0.71) were on DAPT at the time of surgery. Otherwise, tirofiban was used for bridging until 4 h before surgery (TAR group: 65% vs. SV group: 60%; *p* = 0.41).

**Table 1:** Baseline characteristics of the unmatched (left) and matched (right) groups

	Unmatched study population			Matched study population		
Parameter	SV group * n = 296	TAR group * n = 138	*p*-value	SV group * n = 152	TAR group * n = 98	*p*-value
Female gender	85 (29)	24 (17)	0.011	33 (22)	21 (21)	0.96
Body mass index (kg/m^2^)	28 ± 4.7	28 ± 4.8	0.31	28 ± 5.0	28 ± 4.9	0.99
Age, years	71 ± 9.2	59 ± 10	< 0.01	66 ± 9.6	63 ± 9.8	0.08
NSTEMI	202 (68)	95 (69)	0.90	105 (69)	70 (71)	0.69
STEMI	94 (32)	43 (31)		47 (31)	28 (29)	
Coronary artery disease						
1 vessel	11 (3.7)	1 (0.7)	< 0.01	9 (5.9)	1 (1.0)	0.12
2 vessel	42 (14)	25 (18)		21 (14)	18 (18)	
3 vessel	243 (82)	112 (82)		122 (80)	79 (81)	
Diabetes mellitus						
Without insulin	81 (27)	26 (19)	0.054	42 (27)	20 (20)	0.19
With insulin	50 (17)	15 (11)		29 (19)	11 (11)	
Chronic kidney disease						
Stage I (GFR > 89 ml/min)	3 (1.0) 120 (41)	0 31 (22.5)	0.034	2 (1.3) 57 (38)	0 29 (30)	0.13
Stage II (GFR 60-89 ml/min)	75 (25)	10 (7.2)		21 (14)	9 (9.2)	
Stage III (GFR 30-59 ml/min)	8 (2.7) 11 (3.7)	1 (0.7) 1 (0.7)		2 (1.3)	1 (1.0) 1 (1.0)	
Stage IV (GFR 15-29 ml/min)	11 (3.7)	0		6 (3.9)	0	
Stage V (GFR < 15 ml/min)			0.046			0.092
Chronic dialysis						
Arterial hypertension	281 (95)	129 (95)	0.35	145 (95)	90 (92)	0.25
Hypercholesterinemia	195 (66)	92 (67)	0.87	101 (66)	67 (68)	0.75
Cerebral arterial occlusive disease	42 (14)	10 (14)	0.24	21 (14)	11 (11)	0.55
Peripheral arterial occlusive disease						
Fontaine I	6 (2.0)	1 (0.7)	0.078	5 (3.2)	1 (1.0)	0.46
Fontaine II	31 (10)	11 (8.0)		17 (11)	10 (10)	
Fontaine III	5 (1.7)	1 (0.7)		1 (0.7)	1 (1.0)	
Fontaine IV	7 (2.4)	1 (0.7)		2 (1.3)	1 (1.0)	
Chronic obstructive pulmonary disease	32 (11)	11 (8.0)	0.36	16 (11)	8 (8.2)	0.54
PCI before CABG	32 (11)	20 (14)	0.72	12 (7.9)	12 (12)	0.36
Preoperative LVEF						
<20%	21 (7.4)	5 (3.7)	0.047	10 (6.9)	4 (4.2)	0.18
20–30%	27 (9.5)	4 (3.0)		8 (5.6)	3 (3.2)	
31–50%	99 (35)	45 (33)		57 (40)	29 (31)	
>50%	137 (48)	81 (60)		69 (48)	59 (62)	
EuroSCORE II	7.2 ± 8.1	3.4 ± 4.6	**< 0.01**	5.3 ± 6.1	4.8 ± 5.3	0.14

Abbreviations: *CABG* coronary artery bypass grafting, *GFR* glomerular filtration rate, *LVEF* left-ventricular ejection fraction, *NSTEMI* non-ST-segment elevation myocardial infarction, *PCI* percutaneous coronary intervention, *SV* saphenous vein grafts, *STEMI* ST-segment-elevation myocardial infarction, *TAR* total arterial revascularization

^a^Continuous variables: mean ± SD; categorical variables: *n* (%)

### Intraoperative data

**Fig. 1:**
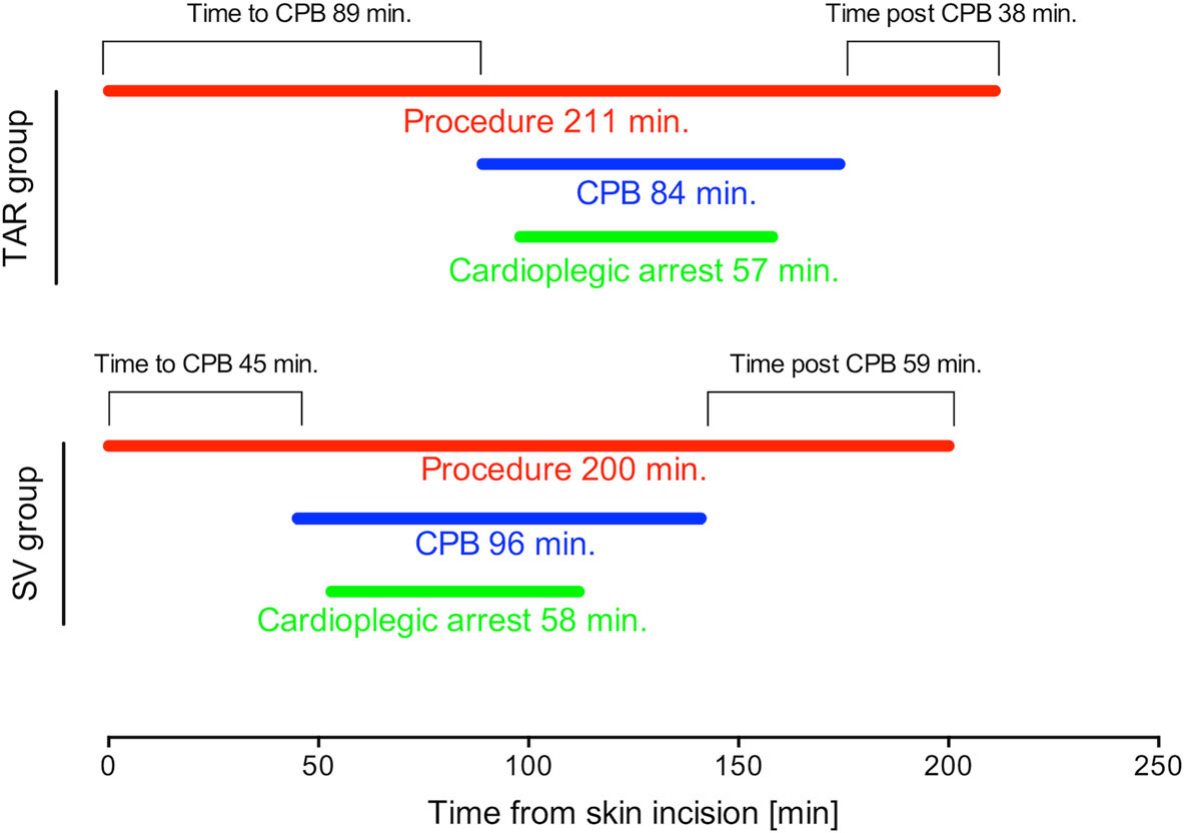
Time course of surgical procedures with either total arterial revascularization or combination of one internal mammary artery and saphenous vein grafts. Abbreviations: CPB: cardiopulmonary bypass; SV: saphenous vein grafts; TAR: total arterial revascularization

The procedures were conducted at a median of 72 h after symptom onset by eight surgeons with a mean experience of 6.5 ± 4.8 years since board certification. Surgeon experience differed significantly between the groups (TAR group: 7.2 ± 4.8 years vs. SV group: 6.0 ± 4.6 years; *p* = 0.042). In 86% of TAR procedures, BIMA grafting was applied, and 14% of patients received a combination of IMA and radial artery grafts. The total procedural times (TAR group: 211 ± 54 min vs. SV group: 200 ± 52 min; *p* = 0.46) did not differ significantly between the groups. The time on cardiopulmonary bypass (CPB) was significantly shorter in the TAR group (84 ± 36 min vs. 96 ± 43 min; *p* = 0.048) and the duration of cardioplegic arrest was similar in the two groups (TAR group: 57 ± 20 min vs. SV group: 58 ± 21 min; *p* = 0.79); however, the distribution of these phases was different, with a longer time to CPB in the TAR group (89 ± 20 min. vs. 45 ± 31 min.; *p* = 0.04) (Fig. 1). This discrepancy can be explained by procedural differences, as the harvesting of both IMA is performed sequentially, whereas the preparation of one IMA and vein grafts is usually carried out simultaneously. Interestingly, the time from the end of CPB to skin closure was significantly reduced in the TAR group (36 ± 10 min vs. 59 ± 15 min, *p* = 0.042). The number of coronary anastomoses did not differ between the groups, and complete revascularization was achieved in 99% (SV group) and 97% (TAR group; *p* = 0.69), respectively (Table 2).

**Table 2:** Preoperative and intraoperative data

Parameter	SV group * n = 152	TAR group * n = 98	*p*-value
Antiplatelet therapy			
ASA	137 (93)	86 (91)	0.44
Ticagrelor	9 (6.1)	10 (11)	0.20
Prasugrel	3 (2.0)	3 (3.2)	0.57
Clopidogrel	44 (30)	20 (22)	0.16
DAPT	51 (34)	32 (33)	0.71
Tirofiban	90 (60)	61 (65)	0.41
Vitamin K antagonists	6 (4.1)	1 (1.0)	0.082
Time interval symptom onset to operation (h)**	72 ± 5.1	72 ± 5.3	0.85
Grafts			
LIMA	147 (97)	98 (100)	**< 0.001**
RIMA	2 (1.3)	84 (86)	
Radial artery	0	18 (18)	
Saphenous vein	152 (100)	0	
Coronary anastomoses			
Total	3.8 ± 1.1	3.6 ± 1.0	0.11
Arterial grafts	1.5 ± 0.6	3.6 ± 1.0	**< 0.001**
Venous grafts	2.3 ± 1.0	0	**< 0.001**
Target vessels			
LAD	151 (99)	98 (100)	0.42
RCX	132 (89)	87 (89)	0.59
RCA	116 (76)	80 (82)	0.32
Complete revascularization [n; %]	149 (99)	95 (97)	0.69

Abbreviations: *CABG* coronary artery bypass grafting, *DAPT* dual antiplatelet therapy, *LAD* left anterior descending artery, *LVEF* left ventricular ejection fraction, *NSTEMI* non-ST-segment elevation myocardial infarction, *PCI* percutaneous coronary intervention, *RCA* right coronary artery, *RCX* Ramus circumflexus, *SV* saphenous vein grafts, *STEMI* ST-segment-elevation myocardial infarction, *TAR* total arterial revascularization

^a^Continuous variables: mean ± SD; categorical variables: *n* (%)

^b^Median ± SD

**Fig. 2:**
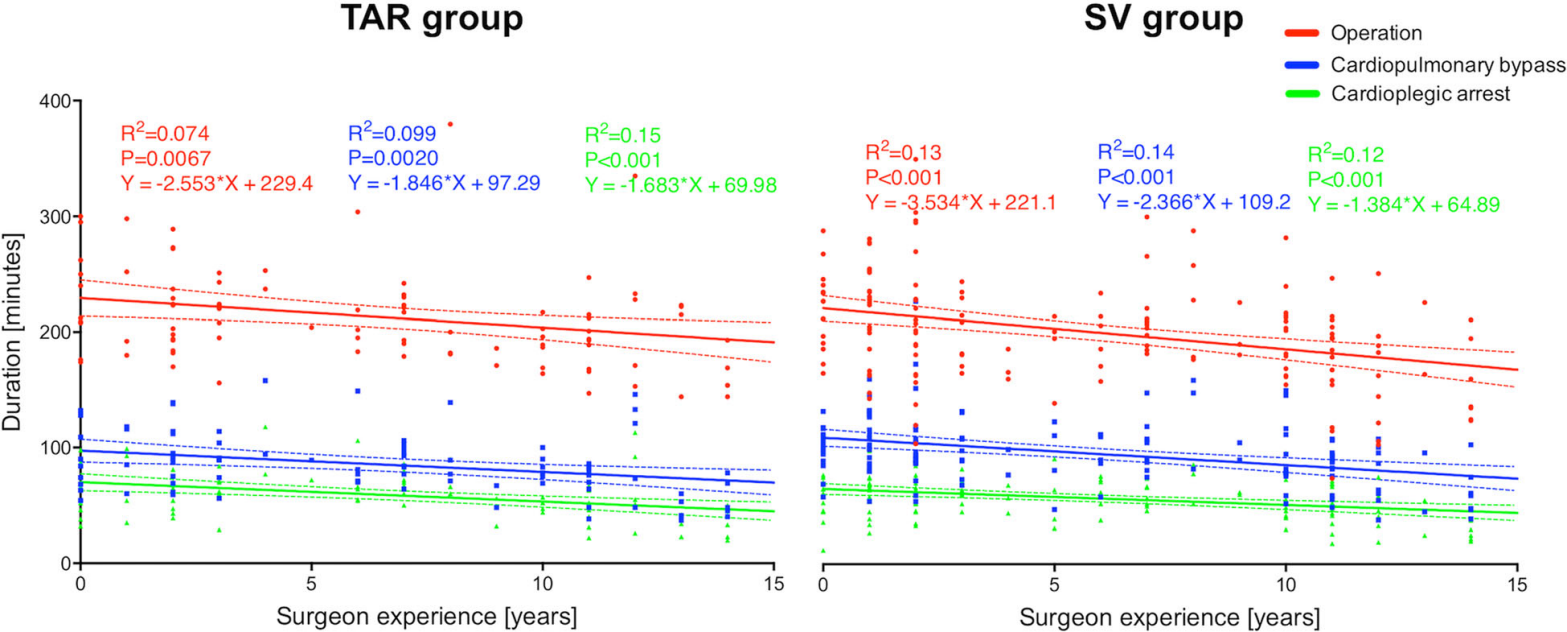
Correlation between surgeon experience and duration of the operation, cardiopulmonary bypass time, and cardioplegic arrest time. Left: Patients who underwent total arterial revascularization; Right: Patients who underwent revascularization with a combination of one internal mammary artery and saphenous vein grafts. Abbreviations: SV: saphenous vein grafts; TAR: total arterial revascularization

The surgeon’s experience had a significant inverse correlation with the total duration of the procedure (2.9 min per year of experience), cardiopulmonary bypass time (2.3 min per year of experience), and cardioplegic arrest time (1.5 min per year of experience). There were no significant differences in this relationship between TAR and SV groups (Fig. 2).

### Perioperative outcomes

**Fig. 3:**
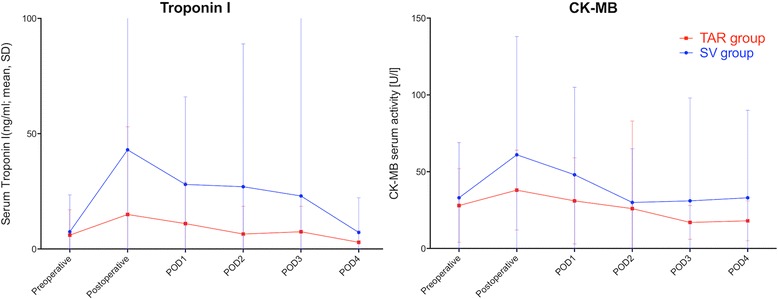
Cardiac injury parameters measured until postoperative day 4. Abbrevisations: CK-MB: Creatine kinase – isoform MB; POD: postoperative day; SV: saphenous vein grafts; TAR: total arterial revascularization

Fifty-seven percent of TAR group patients received erythrocyte transfusion compared with 76% of SV group patients (*p* = 0.001). Platelet transfusion occurred in 36% (TAR group) and 37% (SV group; *p* = 0.86), respectively. Fresh frozen plasma was transfused in 22% of TAR group patients and 30% of SV group patients (*p* = 0.21). In those patients who received transfusions, the median amount of transfused erythrocyte units was higher in the SV group compared with the TAR group (2 vs. 1 unit; *p* = 0.041). The amounts of platelet and fresh frozen plasma transfusions were comparable in both groups (Table 3). The rate of re-explorations due to bleeding was slightly lower in the TAR group than in the SV group (3.1% vs. 5.9%; *p* = 0.30) but this was not statistically significant. Interestingly, surgical revisions for sternal wound healing impairment were not significantly increased in the TAR group (4.0%) compared with in the SV group (2.6%; *p* = 0.52). Serum levels of troponin I and creatine kinase-isoform MB (CK-MB) increased postoperatively, which was followed by a decline until postoperative day 4. The biomarker levels of the TAR group were slightly lower than those of the SV group, but this difference was not statistically significant (Fig. 3). Postoperative intermittent atrial fibrillation occurred less frequently in the TAR group than on the SV group (10% vs. 19%, *p* = 0.059). Acute kidney injury occurred similarly in both groups. Duration of invasive ventilation and rates of tracheostomies for long-term ventilation were comparable in both groups. Consecutively, the median durations of postoperative intensive care unit stay and postoperative hospitalization were similar in both groups. Other postoperative data were comparable between the groups (Table 3).

**Table 3:** Perioperative outcomes

Parameter	SV group * n = 152	TAR group * n = 98	*p*-value
Transfusions			
Erythrocytes			
Rate	116 (76)	56 (57)	< 0.001
Amount (units)**	2 ± 2.6	1 ± 2.7	0.041
Platelets			
Rate	56 (37)	35 (36)	0.86
Amount (units)**	0 ± 1.0	0 ± 0.90	0.33
Fresh frozen plasma			
Rate	45 (30)	22 (22)	0.21
Amount (units)**	0 ± 2.0	0 ± 2.0	0.46
Re-thoracotomy for bleeding	9 (5.9)	3 (3.1)	0.30
Sternal wound healing impairment requiring surgical revision	4 (2.6)	4 (4.0)	0.52
Superficial	2 (1.3)	3 (3.0)	
Deep	2 (1.3)	1 (1.0)	
Duration of invasive ventilation (hours)**	14 ± 58	10 ± 73	0.61
Postoperative tracheostomy	11 (7.2)	5 (5.1)	0.30
New onset atrial fibrillation	29 (19)	10 (10)	0.059
Stroke (>Rankin1)	3 (2.0)	0	0.1
Acute kidney injury			
KDIGO I	59 (40)	32 (33)	0.17
KDIGO II	11 (7.5)	4 (4.1)	
KDIGO III	6 (3.9)	5 (5.1)	
Postoperative dialysis	6 (3.9)	5 (5.1)	0.69
Postoperative length of ICU stay (hours)**	73 ± 81	46 ± 93	0.28
Postoperative length of hospital stay (days) **	10 ± 4.5	10 ± 3.4	0.58
30-day all-cause mortality	6 (4.5)	3 (3.4)	0.68

Abbreviations: *ECLS* extracorporeal life support, *KDIGO* Kidney disease: improving global outcomes, *SV* saphenous vein grafts, *TAR* total arterial revascularization

^a^Continuous variables: mean ± SD; categorical variables: *n* (%)

^b^Median ± SD

### Mortality and long-term follow up

**Fig. 4:**
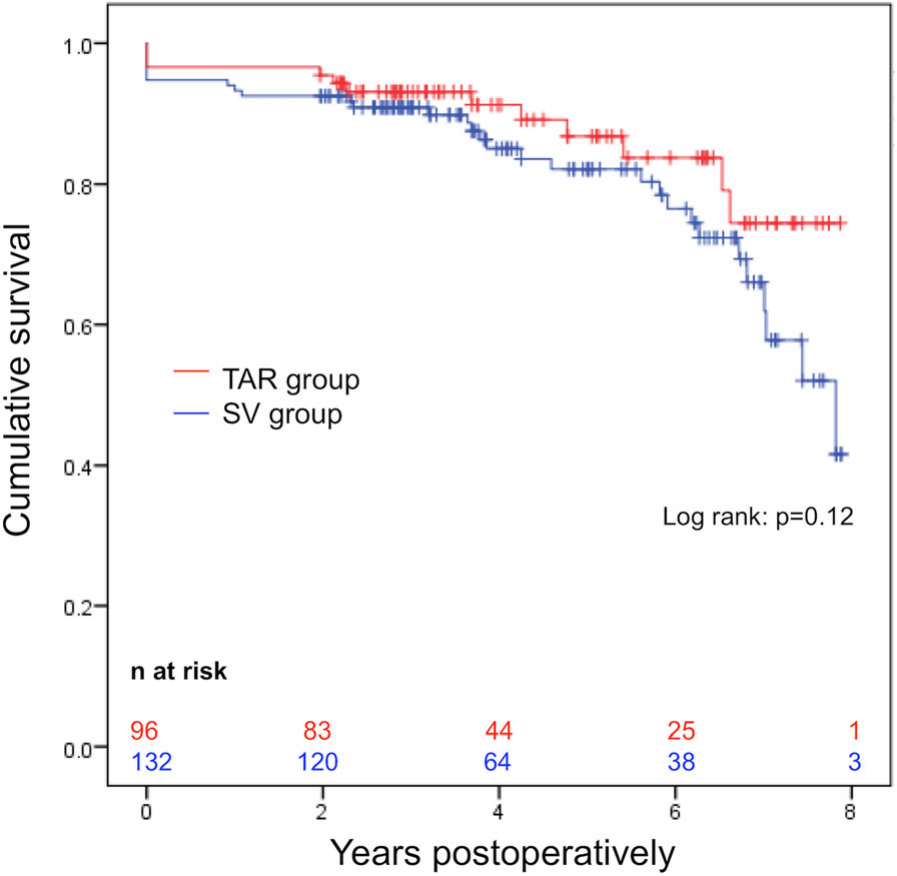
Kaplan-Meier analysis comparing survival of patients who underwent total arterial revascularization or revascularization with a combination of one internal mammary artery and saphenous vein grafts. Abbreviations: TAR: total arterial revascularization; SV: saphenous vein grafts

Mortality at 30 days postoperatively was 4.5% in the SV group and 3.4% in the TAR group (*p* = 0.68).. Further follow-up was complete for 92% of patients with a median follow-up time of 3.7 ± 2.5 years. Kaplan-Meier estimation of survival showed a tendency for improved survival in the TAR group (log-rank *p* = 0.12) with survival curves beginning to diverge from 4 years postoperatively onwards. The overall survival probability at 7 years postoperatively was 75% in the TAR group and 62% in the SV group, respectively (Fig. 4). Symptom-driven repeat coronary angiography was reported by 17% of patients in the TAR group compared with 21% of patients in the SVG group (*p* = 0.45). Redo-CABG was performed in 2 patients (1.3%) in the SV group and 1 patient (1.0%) in the TAR group (*p* = 0.64).

## Discussion

The main finding of this analysis is that CABG using TAR is feasible in patients with AMI as it provides revascularization quality and patient safety like that of CABG using a combination of IMA and SV without increasing the time required for revascularization. Perioperative outcomes did not differ significantly between the groups. Bleeding complications and transfusion requirements were not higher after TAR than after revascularization using IMA/SV; in contrast, the proportion of patients who did not receive any red blood cell transfusion was higher in the TAR group. Postoperative atrial fibrillation was less frequent in the TAR group, possibly due to reduced red blood cell transfusion as demonstrated by previous studies [18, 19]. Nevertheless, if transfusions were necessary, the amount of transfused erythrocyte units was rather high. This might be explained by the high rate of patients with DAPT at the time of surgery [11].

The mean time of surgeon experience was slightly higher in the TAR group, probably reflecting that more experienced surgeons tend to perform this more challenging technique in urgent or emergent clinical settings. Moreover, the increased surgeon experience in the TAR group might result in better surgical results, although recent data did not confirm this assumption for CABG procedures [20–22]. Surprisingly, analysis of procedural duration revealed that the total duration of the surgical procedures involving total arterial CABG and CABG using vein grafts were similar. Our data show that the surgeon’s experience has a significant influence on the duration of the procedure but that the amount is of questionable relevance. The longer phase of graft preparation in the TAR group was balanced by a shorter post-CPB phase in the TAR group. The reduction in reperfusion time and post-CPB time might be partly explained by the greater experience of the surgeons involved, leading to more efficient management at the end of the operation; however, the shorter time could additionally be explained by more rapid bypass graft function of arterial grafts compared with vein grafts, possibly resulting in quicker hemodynamic stabilization. Data on flow properties of arterial bypass grafts compared those of with vein grafts in the immediate intraoperative phase are limited: Spence et al. showed in a canine model that mammary artery graft flow is not impaired by competitive flow from the native vessel [23]. As competitive flow from either the native vessel or collaterals is frequently observed in the early and late postoperative phase, resilience of the grafts may influence their immediate and long-term function [24]. Concerning the immediate function, Weber et al. described improved intraoperative pulsatility indices and a tendency for reduced perioperative myocardial infarctions when using IMA grafts compared with vein grafts [25]. We cannot substantiate our assumption of improved immediate bypass graft function, as flow measurements were not routinely carried out at our institution. Furthermore, although the postoperative increase in serum levels of cardiac biomarkers was somewhat less in the TAR group than in the SV group (possibly reflecting reduced cardiac injury resulting from improved bypass function), this difference was not statistically significant.

We were also surprised to observe that the most technically challenging phase of the procedure, the completion of the coronary anastomoses during cardioplegic arrest, required the same amount of time in the two groups, which is not in keeping with the reluctance to perform TAR due to more difficult and prolonged completion of coronary anastomoses. In fact, the present data should encourage surgeons to commit themselves early to TAR concepts, as these are feasible without loss of time in experienced hands.

Data from the postoperative follow-up period of up to 7 years did not show significant differences in survival between the groups; however, there was a tendency for improved survival in the TAR group from 4 years onwards. The rate of reported symptom-driven repeat coronary catheterizations were non-significantly lower in the TAR group. Unfortunately, there is no information available about the results of these coronary catheterizations and interventions performed. Redo-CABG occurred similarly in both groups. Previous studies have demonstrated that differences in graft patency between SV and IMA grafts become evident only after 4–8 years [26, 27, 28]. A survival benefit after TAR in the mid- or long-term has been shown in pooled analyses [29, 30]. Our observation is in accordance with the recently published work by Taggart et al. showing no significant survival benefit after bilateral IMA versus single IMA grafting after 5 years [31]. A longer-term follow-up of the patients will be required to confirm these observations.

Several limitations of this study should be mentioned. First, patients with LCOS prior to surgery were excluded from this analysis, as CABG in these patients frequently does not follow the standardized sequence of operative steps. These patients are often placed on CPB prior to graft harvesting, and BIMA preparation is all but ruled out in these emergency situations, and hence we did not consider these exceptional, very individual situations to be suitable for a generalizable analysis. Therefore, the results of this study cannot be applied to patients who present with LCOS before CABG. Second, CPB with cardioplegic arrest was used in all procedures. Alternative approaches include off-pump CABG or on-pump CABG with beating heart [32–34], which might reduce injury and inflammation associated with CPB and cardioplegic arrest. CPB with cardioplegic arrest, however, provides hemodynamic stability during the procedure with optimized exposure for accurate anastomosing. To date there are no data available showing superiority of one strategy over the other.

Although the logistical and technical aspects (procedural times, completeness of revascularization) of our study are not likely to be biased by the study design, outcome data of this retrospective, propensity-matched analysis should be considered with caution. Unknown confounders might reduce comparability of the groups and bias outcome data.

## Conclusion

TAR should be considered the standard of care in hemodynamically stable patients with AMI undergoing CABG, as it is equally safe and rapid compared with the use of combinations of IMA and vein grafts. Reluctance to apply TAR in these patients due to fear of protracted revascularization and bleeding complications is no longer justified. Long-term outcome may be improved after TAR, but these observations remain to be confirmed in a longer-term study.

## Abbreviations

AMI: Acute myocardial infarction
BIMA: Bilateral internal mammary artery
CABG: Coronary artery bypass grafting surgery
CK-MB: Creatine kinase, isoform MB
CPB: Cardiopulmonary bypass
LCOS: Low cardiac output syndrome
NSTEMI: Non-ST-segment elevation myocardial infarction
OR: Odds ratio
SD: Standard deviation
STEMI: ST-segment-elevation myocardial infarction
SV: Saphenous vein grafts
TAR: Total arterial revascularization
